# Trends in the decapod crustacean community at the southernmost estuary of the Atlantic coast of Europe

**DOI:** 10.1038/s41598-023-50049-9

**Published:** 2023-12-21

**Authors:** Enrique González-Ortegón, Gustavo F. de Carvalho-Souza, Cesar Vilas, Francisco Baldó, Jose A. Cuesta

**Affiliations:** 1 Institute of Marine Sciences of Andalusia, Spanish National Research Council (ICMAN-CSIC), Puerto Real, Spain; 2 Associate Research Unit “Blue Growth”, Spanish National Research Council (CSIC) - Andalusian Institute of Agricultural and Fisheries Research and Training (IFAPA), Cadiz, Spain; 3 Universidad de Cádiz, Departamento de Física Aplicada, Instituto Universitario de Investigación Marina (INMAR), Campus de Excelencia Internacional/Global del Mar (CEI·MAR), 11519 Puerto Real, Cádiz Spain; 4 Instituto de Investigación y Formación Agraria Pesquera (IFAPA), Centro El Toruño, Camino Tiro de Pichón S/N, 11500 El Puerto de Santa María, Spain; 5 Spanish Institute of Oceanography, C.O. de Cádiz (IEO-CSIC), 11006 Cadiz, Spain

**Keywords:** Community ecology, Environmental impact

## Abstract

Climate change may enhance the establishment of introduced species, as well as the poleward shift in distribution of numerous species over decades. Long-term research and monitoring of an ecosystem at the southernmost point of the Atlantic coast of Europe should be an important priority in order to detect and understand trends in species composition and the related environmental changes. The Guadalquivir estuary (South West Spain) is more likely to suffer the exacerbated effects of climate change due to its location in the Mediterranean-climate zone. The long-term data set between 1997 and 2006 has allowed us to analyse the variability of the natural and anthropogenic stressors. The mean interannual dissimilarity of the estuarine fauna (Bray–Curtis dissimilarity index) has showed important differences throughout the years, and the species that most contributed to these differences were the exotic species capable of completing their life cycles. This long-term monitoring of the estuarine community has allowed us to anticipate future events and ecological risk assessment in European waters.

## Introduction

The ecological importance of decapod crustaceans has been previously shown in the numerous studies on the composition, distribution and biology of their species, mainly those of commercial interest, that have been carried out in different European estuaries^1–4^. Similarly, the shrimp fishery in estuaries such as the Guadalquivir estuary of the Atlantic coast of Europe^5^ and the prawn fishery in the Gulf of Cadiz^6^ indicate the value of these species as a resource for fishermen in the area.

Among the species of a decapod crustacean community, benthic species are the most abundant in estuaries and shallow bays of the northeastern Atlantic coast^7^. The use of tidal currents, mainly by larval marine species, to penetrate estuaries and the high estuarine production, due to the natural discharge of nutrients by rivers^8^, make these shallow and muddy environments suitable for the establishment of benthic species, such as *Crangon crangon* (Linnaeus, 1758).

The community structure of decapod crustaceans in an ecosystem are affected and driven by a range of biotic and abiotic factors that create biotic patterns through their interactions. In temperate estuaries, marked seasonality is a widespread characteristic of their aquatic communities^1,9,10^, being mainly water temperature and salinity, and, to a lesser extent, turbidity, substrate type, substrate cover and water quality, the main factors influencing species abundance and spatiotemporal distribution^11^. Since the seasonal pattern of salinity in an estuary is, in principle, determined by rainfall and consequent river flow, in most large European estuaries, temperature and salinity follow a similar seasonal pattern: with summer maxima and winter minima, although there may be slight shifts in the seasonal evolution of both variables^7,12–14^. This fact makes it difficult to separate the role of temperature and salinity in structuring aquatic communities in estuaries. In addition, the spatial and temporal patterns observed in the distribution of species may be the result of the indirect influence of river water on other physicochemical variables, such as dissolved oxygen^15^.

In general, temperature appears to have a major influence in determining the entrance of many marine species in estuaries, the breeding periods of estuarine species, as well as their growth and reproductive rates, and temporal changes in their abundance and biomass^2,16,17^. In contrast, salinity is primarily responsible for the spatial distribution of organisms within the estuary, but retention of freshwater discharge from rivers into estuaries is associated with changes in salinity and water quality, and "unexpected" reductions in salinity in the dry and warmer period affect the composition and dynamics of estuarine communities^18^.

In addition to the marked seasonal pattern of environmental conditions in European estuaries, they often show important interannual variations in their conditions and, consequently, in their aquatic communities. For example, in the case of fish species in the Guadalquivir estuary, a spatial distribution pattern has been observed in which the number of species and their density gradually decreases upstream^1^. However, the degree of penetration, density attained and period of permanence of each species in the different areas of the estuary varies considerably from one year to another^19^. These interannual variations seem to be closely related to the longitudinal displacement of the salinity gradient due to the input of freshwater discharges of each year^1^. In this sense the use of long-term ecological research (LTER) are more relevant to discern the importance of any factors under the complex and the variability of these ecosystems.

Other factors which can alter the structure of a community are exotic species. The increase of the abundance and biomass of a new species in an ecosystem may also alter biotic conditions. Exotic species are increasingly becoming the focus of research and have been identified as a component of human-induced global change^20^. Many estuaries are subject to intense international maritime traffic, which constitutes a pathway for the entry and dispersal of allochthonous aquatic species^21,22^. For instance, in the Guadalquivir River estuary, there are several examples of introduced species, such as the American mud crab *Rhithropanopeus harrisii* (Gould, 1841), the Chinese mitten crab *Eriocheir sinensis* H. Milne Edwards, 1853, or the oriental shrimp *Palaemon macrodactylus* Rathbun, 1902^23^, which in just 8 years has become one of the dominant species of decapod crustaceans in many other European estuaries. This fact is not over, since in recent years the presence of a new species of decapod crustacean, *Callinectes sapidus* Rahbun, 1896, has been detected in the area^24,25^. Undoubtedly, the entry of a new element into a system, such as a species, could causes changes in the natural structure of the system. The complexity of the system becomes somewhat greater and, although its diversity increases in an initial phase, the changes that the exotic species can cause in the structure of the original community of the estuary can lead to an alteration of the ecological processes and balances that are specific to it.

The general objective of this work is to study the nektonic community of decapod crustaceans of the Guadalquivir river estuary, and to know the interannual variability due to the effects of the entry of an exotic species on the community structure. For this purpose, we propose to characterize, over time, the environmental conditions of the estuary, as well as the composition and structure of the nektonic community of the decapod crustaceans that inhabit it, and to establish the relationship between both groups of variables.

The hypothesis is that the temporal distribution of decapod crustacean species is largely determined by the environmental conditions of the estuary, especially by the salinity gradient and water temperature, and by the entry of exotic species.

## Results

### Composition of decapod crustacean community

During the study period (June 1997–December 2006), a total of 35 species of decapod crustaceans ascribed to 23 families were collected in the Guadalquivir estuary, with the Palaemonidae family standing out for having the largest number of species in the estuary (Table 1). From these, only 3 marine migrants (MM) and 3 estuarine species (ES) were regularly present in the estuary. In turn, a high number (29) of straggler species (SS), mostly marine, was collected, but these only added up to a 2.1% of the total abundance, on average. In this study period, four taxa were allochthonous. These species were *Procambarus clarkii* (Girard, 1852)*, R. harrisii, E. sinensis and P. macrodactylus.*

**Table 1:**
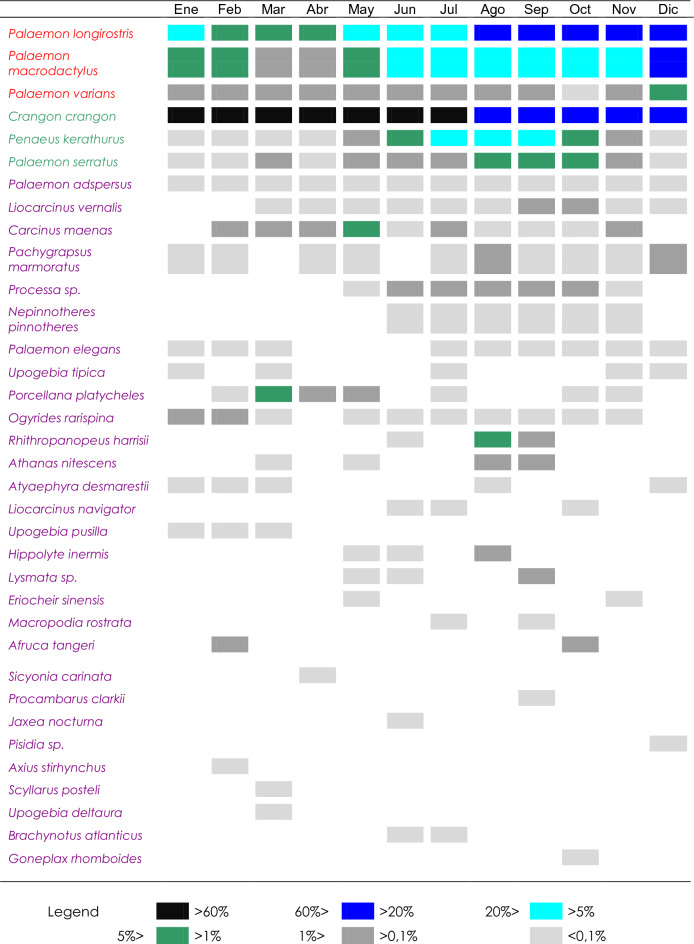
Mean monthly dominance of decapod crustacean species caught in the Guadalquivir estuary during the entire study period (June 1997–December 2006), in percent. Species are ordered from highest to lowest mean constancy in the estuary and by ecological categories (Estuarine species (red font), Seasonal or marine migrants species (green font), Accidental or straggler species (violet font)).

**Figure 1:**
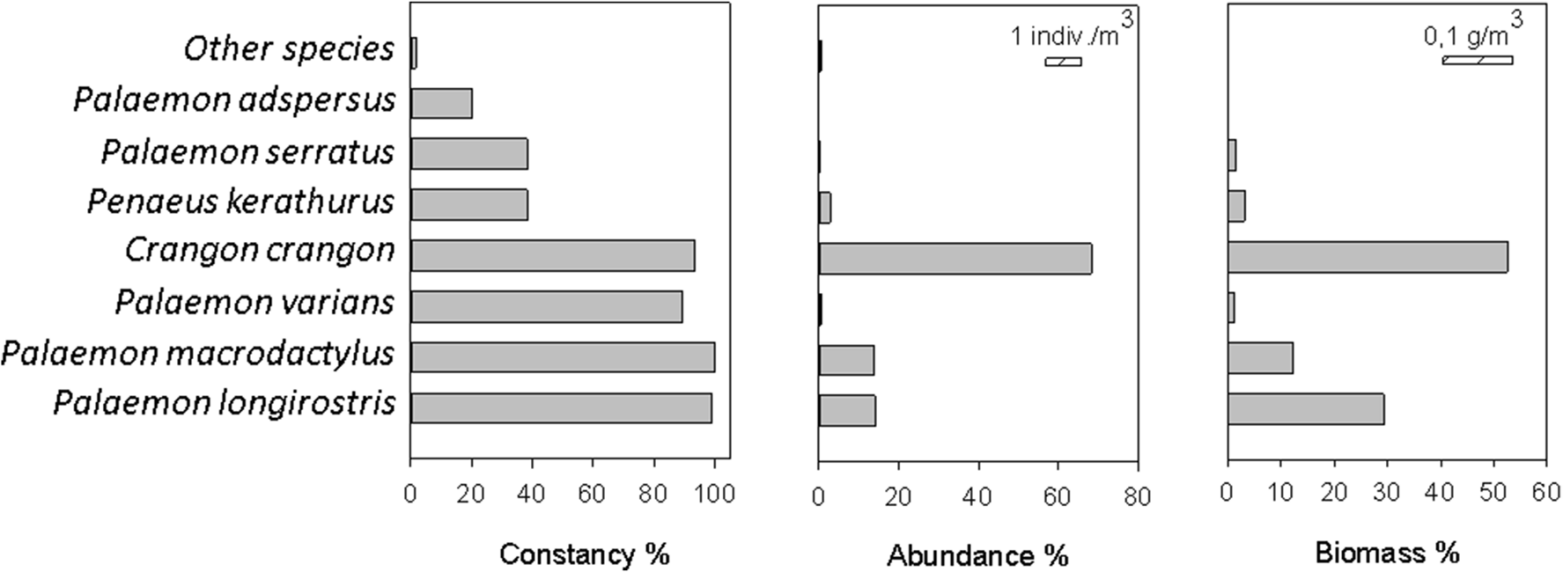
Mean constancy, abundance, and biomass for the entire study period (June 1997–December 2006) in percent of the decapod crustacean species in the Guadalquivir estuary. The horizontal striped bars in the abundance and biomass graphs represent the scale in absolute values.

In terms of abundance and biomass (dominance) by species, *C. crangon* was the species with the highest mean values (68.4% abundance and 50.8% biomass), followed by *Palaemon longirostris* H. Milne Edwards, 1837 (13.2% and 32.6%, respectively), *P. macrodactylus* (9.6% and 8.1%), *Penaeus kerathurus* (Forskål, 1775) (5.8% and 4.0%), *Palaemon serratus* (Pennant, 1777) (0.6% and 3.6%), *Palaemon varians* Leach, 1814 (0.3% and 0.6%) and, finally, the rest of the species with very low dominances (Fig. 1).

When the dominance of the different species was analysed monthly, it was observed that *C. crangon* was the species that showed the highest values all year round (38–95%), except in October and November when *P. longirostris* predominated (33–37%). In addition to these two species, *P. macrodactylus* and *P. kerathurus* also showed relatively high dominance values in August and September (Table 1).

The presence of the estuarine species, *P. longirostris* and *P. macrodactylus*, was high throughout the estuary as they complete their life cycle within this ecosystem, and of *P. varians*, which lives mainly in the canals and marshes near the estuary, hence its relatively low abundance in the main channel.

Seasonal species are coastal marine species that enter the estuary mainly between spring and summer, such as *P. kerathurus*, *P. serratus* and *C. crangon*.

Straggler species showed generally very low constancy and dominance throughout the estuary. Within this category, there are some freshwater species such as *Atyaephyra desmarestii* (Millet, 1831), *P. clarkii* and *R. harrisii*, the freshwater and migrant crab *E. sinensis*, and mostly marine species.

### Diversity and dominance of the decapod community

**Figure 2:**
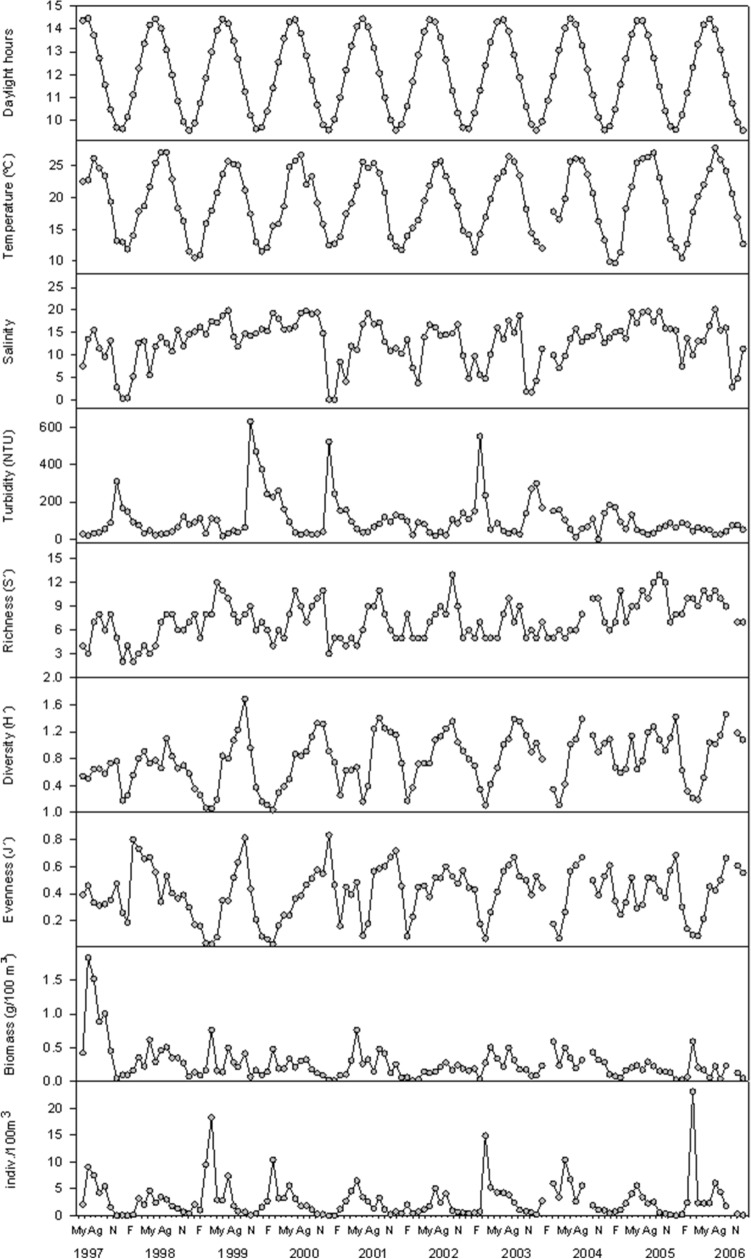
Day length, water temperature, salinity, mean turbidity, specific richness, diversity (Hʹ), evenness (Jʹ), biomass and mean abundance of the decapod crustacean community in the Guadalquivir estuary from June 1997 to December 2006.

From June 1997 to December 2006, the Shannon–Wiener diversity and equitability indices, and the abundance and mean biomass of the decapod crustacean community showed considerable temporal variations, with a clear seasonal component; while the specific richness, which also showed significant temporal variations, ranging from 2 to 13 species per season, did not show a clear seasonal pattern (Fig. 2).

**Figure 3:**
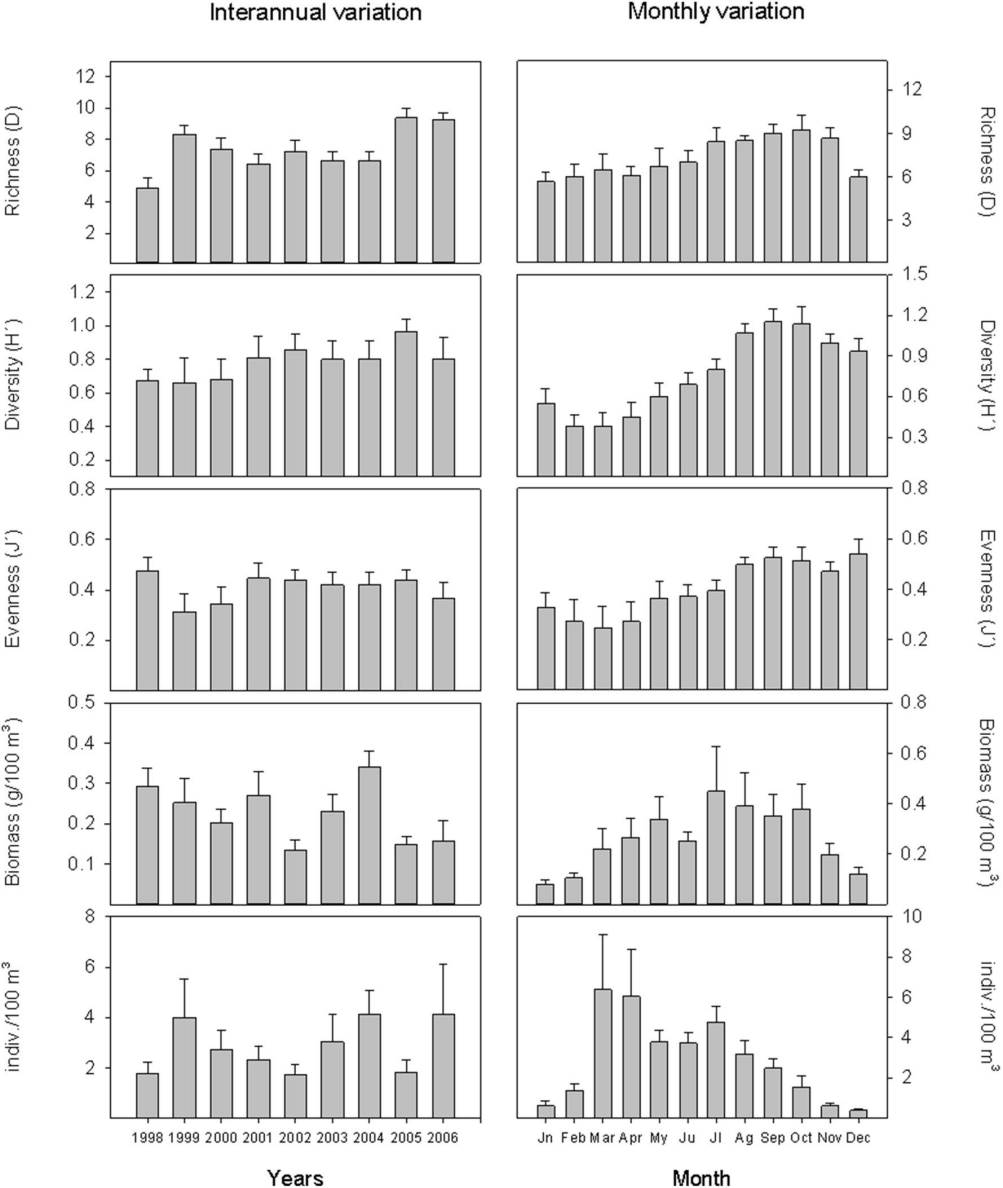
Interannual and monthly variations of specific richness, diversity (Hʹ), evenness (Jʹ), biomass and mean abundance of the decapod crustacean community in the Guadalquivir estuary between June 1997 and December 2006.

In general, diversity and equitability showed all year peaks of similar magnitude in autumn, increasing their values gradually between winter and autumn; specific richness, on the other hand, showed greater seasonal variability (Fig. 3). More specifically, specific richness, diversity and equitability presented their lowest mean values in winter (January–March) and the highest mainly in autumn (September–December). On the other hand, it should be noted that the highest value of the diversity index occurred in October 1999, coinciding with the entry of the new exotic species *P. macrodactylus* into the estuary in that same year (Fig. 2).

Over this time series, the total biomass of decapod crustaceans presented its highest value at the beginning of the study period (1.8 g/100 m^3^ in July 1997), but in terms of abundance, it usually occurred in spring (the highest value was 23 indiv./100 m^3^ in March 2006) (Fig. 2). Regarding the seasonal pattern of mean biomass and mean abundance of the decapod crustacean community, their lowest mean values occurred in January (0.08 g/100 m^3^) and December (0.38 indiv./100 m^3^), respectively; and the highest values occurred in July (0.45 g/100 m^3^), and in March (6.39 indiv./100 m^3^), after a progressive growth, in the case of biomass, and a sharp increase, in the case of abundance (Fig. 3).

As for the interannual variation (Fig. 3), there was no homogeneity in the patterns of change of the different variables. Thus, specific richness was slightly higher in 1999, 2005 and 2006; the diversity index increased slightly between 1998 and 2005; and in the case of equitability, with a lower interannual variation and a mean value of 0.40, it had its lowest value (0.31) in 1999. In the case of biomass, the mean annual values ranged from 0.34 g/100 m^3^ in 2004 to 0.13 g/100 m^3^ in 2002. Abundance also showed some interannual variation, ranging from high mean abundance in 1999, 2004 and 2006 (> 4.0 indiv./100 m^3^) to relatively low mean abundance in 1998 and 2002 (< 2.0 indiv./100 m^3^).

When analysing the correlation between the monthly mean values of these biological variables and the environmental variables during the study period (Table 2), it was observed that the equitability was not significantly correlated with any of the environmental variables. In the case of specific richness, the maximum correlation was with salinity (r = 0.61); in that of the diversity index and biomass it was with temperature (r = 0.35 and 0.54, respectively); and in that of abundance, day length in daylight hours (r = 0.72). In addition, it should be noted that the density of the mysid *Mesopodopsis slabberi* (Van Beneden, 1861) was well correlated with the abundance (r = 0.62) and biomass (r = 0.52) of the decapod crustacean community. On the other hand, turbidity, FSW discharges from Alcalá del Río dam and rainfall were environmental variables that were generally negatively correlated with the biological variables. FSW discharges were most correlated with specific richness (r = − 0.42), while turbidity was most correlated with the diversity index (r = − 0.29) and precipitation with abundance (r = − 0.50) and biomass (r = − 0.36).

**Table 2:** Spearman correlation coefficients (r) between biological variables (specific richness (Sʹ), diversity index (Hʹ), equitativity (Jʹ), abundance (A) and mean biomass (B) of the decapod crustacean community) and environmental variables (number of daylight hours (L), salinity (S), temperature (Tª), water turbidity (Tu), rainfall (P) and volume of water released from the Alcalá del Río dam (D) during the 28 and 9 days prior to the campaign and abundance of *M. slabberi* (M.s)). (*) = pre-Log transformation; n = number of cases; p = significance level.

	L	S	Tª (ºC)	Tu* (UNT)	P_28_* (l/m^2^)	P_9_* (l/m^2^)	D_28_* (hm^3^)	D_9_* (hm^3^)	M.s*
Sʹ									
n	104	104	104	103	103	103	104	104	103
r	0.15	0.61	0.39	− 0.33	− 0.31	− 0.25	− 0.42	− 0.38	0.32
p	> 0.05	< 0.01	< 0.01	< 0.01	< 0.01	< 0.05	< 0.01	< 0.01	< 0.01
Hʹ									
n	104	104	104	103	103	103	104	104	103
r	− 0.05	0.19	0.35	− 0.29	− 0.19	− 0.14	− 0.07	− 0.09	0.26
p	> 0.05	> 0.05	< 0.01	< 0.01	< 0.05	> 0.05	> 0.05	> 0.05	< 0.01
Jʹ									
n	104	104	104	103	103	103	104	104	103
r	− 0.08	− 0.11	0.19	− 0.18	− 0.04	− 0.06	0.16	0.14	0.14
p	> 0.05	> 0.05	> 0.05	> 0.05	> 0.05	> 0.05	> 0.05	> 0.05	> 0.05
B*									
n	104	104	104	103	103	103	104	104	103
r	0.43	0.14	0.55	− 0.34	− 0.36	− 0.31	0.05	0.07	0.52
p	< 0.01	> 0.05	< 0.01	< 0.01	< 0.01	< 0.01	> 0.05	> 0.05	< 0.01
A*									
n	104	104	104	103	103	103	104	104	103
r	0.73	0.31	0.58	− 0.37	− 0.50	− 0.32	0.02	0.10	0.62
p	< 0.01	< 0.01	< 0.01	< 0.01	< 0.01	< 0.01	> 0.05	> 0.05	< 0.01

### Linking environmental conditions to decapod crustacean community

**Figure 4:**
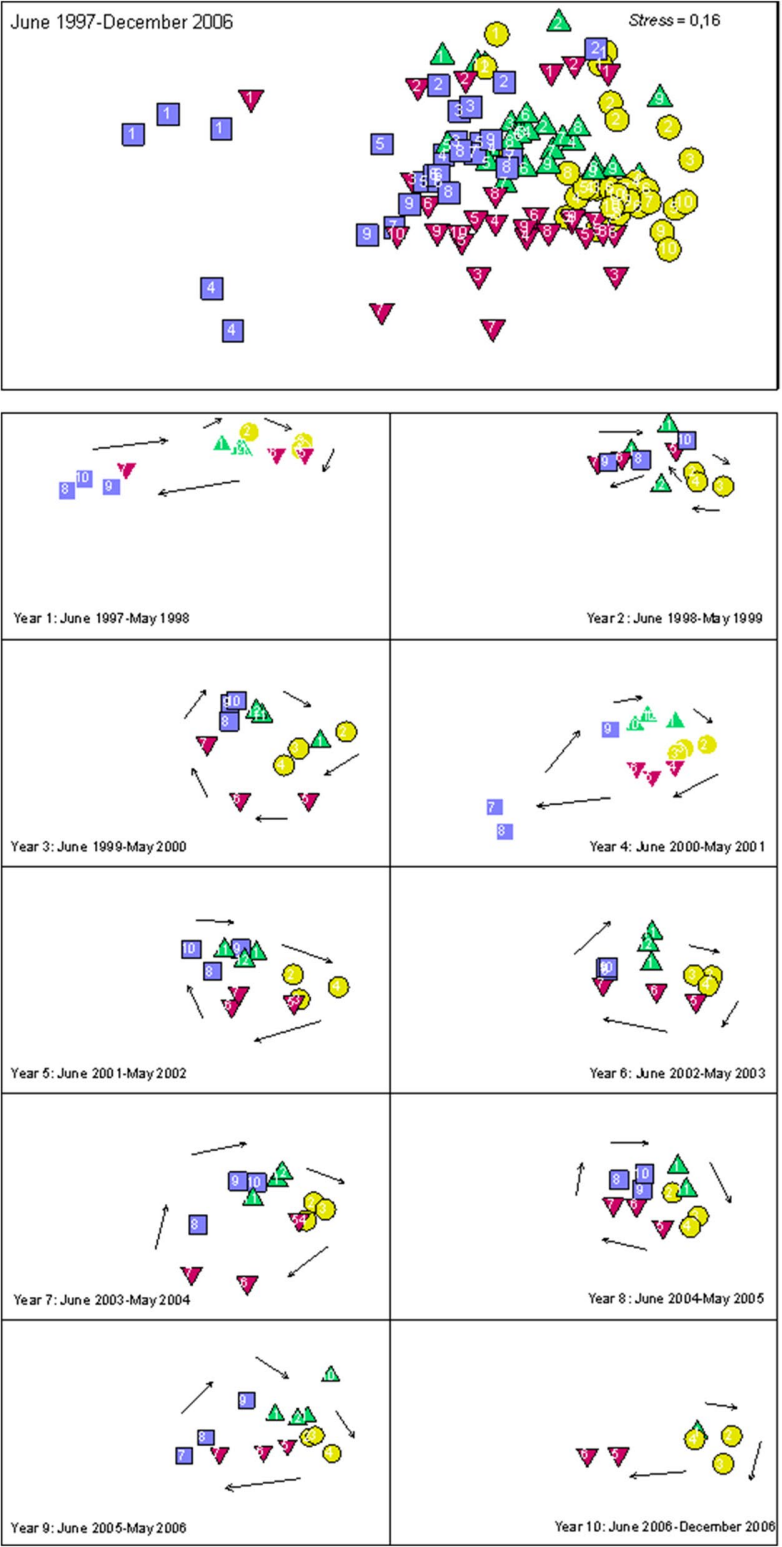
nMDS ordination of the monthly mean values of the abundance of the decapod crustacean community in the Guadalquivir in different years between June 1997 and December 2006 (Bray–Curtis similarity index). Green upper triangle = spring month; Yellow circle = summer month; purple lower triangle = autumn month; blue square = winter month.

The nMDS ordinations indicated a cyclical pattern: summer months were located on the right side, while the winter months were on the left side. This temporal segregation of the samples was more noticeable for the rainy years (Fig. 4, years 1 and 4). On the other hand, springs were generally located in the upper part of the graph and autumns in the lower part. This cyclical pattern was not clear the second year.

In addition, when the mean similarity of the different years was analysed, it was observed that years 1 and 4 (rainy years) presented the lowest values, for which the winter months were located farthest to the left of the nMDS ordination graph. The species that contributed most to the similarity between the different months of each year was *C. crangon*, followed by *P. longirostris*, which contributed most during the first year (Table 3). However, when the mean dissimilarity between the different combinations of years was analysed, it was observed that years 1 and 2 in combination with the rest showed significant differences of particular importance. The species that contributed most to these differences was the exotic species *P. macrodactylus* (Table 3).

**Table 3:** Mean similarity between samples for the same year and dissimilarity between samples from different years in the estuary (SIMPER analysis, Bray–Curtis coefficient). Species contributing at least 10% to the similarity or dissimilarity of years: Cc, *C. crangon*; Pl, *P. longirostris*; Pm, *P. macrodactylus*; Pk, *Penaeus. kerathurus*; Ps, *P. serratus*; Pv, *P. varians*. Significance levels: *p < 0.001, **p < 0.05 and NS: non-significant p > 0.05.

	Years		Value	Species
Similarity	1		64.9	Pl > Cc > Pv
	2		73.1	Cc > Pl
	3		69.8	Cc > Pl > Pm
	4		66.4	Cc > Pl > Pm > Pv
	5		70.8	Cc > Pl > Pm > Pv
	6		73.1	Cc > Pl > Pm > Pv
	7		71.4	Pm > Pl > Cc > Pv
	8		**76.1**	Cc > Pl > Pm
	9		71.1	Cc > Pm > Pl
Disimilarity	1–2	*	36.8	Cc > Pl > Pk > Pv > Ps
	1–3	*	45.8	Cc > Pl > Pm > Pk > Ps
	1–4	*	45.8	Pm > Pl > Cc
	1–5	*	44.1	Pm > Cc > Pl
	1–6	*	44.5	Pm > Cc > Pl > Pk
	1–7	*	45.2	Pm > Cc > Pl > Pk
	1–8	*	41.5	Pm > Cc > Pl
	1–9	*	**49.6**	Cc > Pm > Pl > Ps > Pk
	2–3	*	32.8	Cc > Pm > Pk > Pl > Ps
	2–4	*	38.1	Cc > Pm > Pl > Pk > Ps
	2–5	*	34.9	Pm > Cc > Pk > Pl > Ps
	2–6	*	35.7	Pm > Cc > Pk
	2–7	*	39.7	Pm > Cc > Pk
	2–8	*	32.6	Pm > Cc > Pk > Pl > Ps
	2–9	*	38.1	Pm > Cc > Pk > Ps > Pl
	3–4	NS	32.7	Cc > Pk > Pl > Pm > Ps
	3–5	NS	30.1	Cc > Pk > Pm > Ps > Pl
	3–6	NS	30.5	Pk > Cc > Pm > Ps
	3–7	*	35.2	Cc > Pm > Pk > Ps
	3–8	**	29.2	Cc > Pm > Pk > Pl > Ps
	3–9	NS	31.3	Cc > Pk > Pm > Ps
	4–5	NS	30.7	Cc > Pl > Pk > Pm > Ps
	4–6	NS	30.3	Cc > Pk > Pl > Pm > Ps
	4–7	NS	32.2	Cc > Pm > Pk > Pl
	4–8	NS	28.5	Cc > Pl > Pm > Pk > Ps
	4–9	NS	32.8	Cc > Pk > Ps > Pl > Pm
	5–6	NS	27.2	Pk > Cc > Pm > Ps
	5–7	NS	30.8	Cc > Pm > Pk > Ps
	5–8	NS	27.2	Cc > Pm > Pl > Pk > Ps
	5–9	NS	29.8	Cc > Pk > Pm > Ps
	6–7	NS	28.1	Cc > Pk > Pm
	6–8	NS	26.8	Pk > Cc > Pl > Pm > Ps
	6–9	NS	28.8	Cc > Pk > Ps > Pm
	7–8	NS	28.3	Cc > Pm > Pk > Pl > Pv > Ps
	7–9	**	32.1	Cc > Pk > Ps > Pm
	8–9	*	30.7	Cc > Pl > Pm > Pk > Ps

The highest similarity value for each year and dissimilarity value between years are in bold.

The correlation coefficients between the coordinates of the samples for axis I, and the corresponding values of the environmental variables (Table 4), indicated that water temperature (0.81) showed the best correlation. The rest of the environmental variables had significant correlations with the exception of the FSW discharges from the Alcalá del Río dam. In the case of day length, salinity, temperature and density of *M. slabberi* the correlation was positive; while that of turbidity and precipitation was negative. For axis II, day length was the only variable with a significant correlation, and this was positive (0.32).

**Table 4:** Spearman correlation coefficients between environmental variables and the coordinates of the sampling months for each of the ordination axes (nMDS) of the decapod crustacean community in the global nMDS ordination displaying all monthly sampling points. r = value of the Spearman correlation coefficient; p = significance level. (*) = pre-Log transformation.

	Estuary N = 105	
	Axis I	Axis II
Salinity		
r	0.55	− 0.17
p	< 0.01	> 0.05
Temperature		
r	**0.81**	0.02
p	< 0.01	> 0.05
Daylight hours		
r	0.58	**0.32**
p	< 0.01	< 0.01
Turbidity*		
r	− 0.57	− 0.04
p	< 0.01	> 0.05
Rainfall*		
r	− 0.58	− 0.11
p	< 0.01	> 0.05
FSW discharges*		
r	− 0.16	0.14
p	> 0.05	> 0.05
M. slabberi*		
r	0.71	0.13
p	< 0.01	> 0.05

The Highest correlation coefficients in axis I and II are in bold. Axis I in the nMDS is the horizontal axis (Axis X), and Axis II is the vertical axis (Axis Y).

When the biological and environmental matrix values were correlated (Table 5), it was observed that the density of the mysid *M. slabberi* as prey (0.44) was the variable that best explained the distribution of community abundance samples, followed by temperature (0.38). However, the combination of two variables that best explained the biological matrix was temperature and salinity (0.51).

**Table 5:** Correlation values between the biological similarity matrix and the matrix of estuarine environmental variables (BIOENV analysis, Spearman). (*) = pre-Log transformation.

Salinity	0.343
Turbidity*	0.190
Temperature	0.387
Light	0.264
FSW Discharges*	0.279
Rainfall*	− 0.014
Abundance M. slabberi*	0.445
Best 2, 3 y 4 combinations	
Temperature and Salinity	0.513
Temperature, Salinity and day length (light hours)	0.525
Temperature, Salinity day length and M. slabberi*	0.529

## Discussion

### The decapod crustacean community

The long duration of the sampling period and the fact that samples have been collected monthly have allowed us to learn about the fauna inhabiting an estuary under very diverse environmental conditions, as well as to witness the introduction and establishment of an exotic species of palaemonid, the oriental shrimp *P. macrodactylus*^26^. The estuarine and seasonal marine species (dominant groups of species) are coincident with those established in other European estuaries^9,10,12,27^.

On the other hand, there are species that do not appear in the estuary every year, accidental species. These marine species, although not established within the estuary, increase the number of occasional species collected. Low freshwater input due to the climate and the strong influence of the incoming tidal current that salinizes the lower part of the estuary, allows and facilitates the entry of marine species from the coast, especially planktonic decapod larvae. Similarly, estuarine salinization favoured the incursion of marine species into the estuaries^14^, although they were mostly accidental marine species, with no apparent estuarine requirements and whose presence in the estuaries was irregular^28^. The number of these species is expected to increase in the future as a result of climate change, which will lead to the expansion of the distribution of southern species to higher latitudes^29^.

Although marine species tend to be the most abundant group of species in the Mediterranean estuaries, some estuarine species such as *P. longirostris* also reach high densities (Fig. 1) as in other European estuaries^9,10,12^. All of them, including the benthic species *C. crangon*, have been abundant species in the estuary, both numerically and in terms of biomass, which makes them key species in the functioning of the ecosystem. In addition, due to their strong dominance, these species are also the main responsible for marking the structural variations of the community temporally.

### The intra-annual variability of the community

The decapod crustacean community of the Guadalquivir estuary has shown practically cyclical seasonal changes, with the massive recruitment of *C. crangon* (in spring and, above all, in summer) and the relatively high importance of *P. longirostris* in autumn and winter. These two species are the main contributors to the homogeneity of the community at each time of the year in European estuaries^9,27,30^. That is, between one winter and the following autumn, the decapod crustacean community registers a progressive increase in specific richness and its mean diversity, due to the gradual entry of marine species into the estuary and their subsequent permanence there until the end of the period in which marine species left the estuary (autumn) ^7,12–14^.

Along a year, in regards to the seasons with the greatest variations in community composition and structure, summer and winter, coincided with the most extreme environmental conditions. Basically, in summer there was an increase in the densities of *P. longirostris*, *P. macrodactylus* and *C. crangon*, and *P. kerathurus* and *P. serratus* appeared, while in winter the opposite occurred. Therefore, variations in the densities of some species (such as *P. longirostris*, *P. macrodactylus* and *C. crangon*) and migrations between the estuary and the sea of others (such as *P. kerathurus* and *P. serratus*), are partly responsible for the annual cyclical variations. However, temperature is often the determining factor in the reproductive cycle of many of these species and, consequently, the ultimate determinant of these seasonal changes^14,30^.

### The Interannual variability of the community

The species *P. longirostris* and *C. crangon* presented the highest biomass (32% and 47% of the total mean biomass of nektonic decapod crustaceans, respectively) in the sampled area, as in most of the European Atlantic estuaries^10,31^. These species make them, functionally the most important decapod crustacean species, in energy flows in this type of ecosystem. However, the high growth rate of the allochthonous species *P. macrodactylus* during the 10 years of study since the first two females captured in January 1999 (its first detection in the estuary), also makes it a component of some importance in this ecosystem (7.5% of the total biomass of decapod crustaceans). This non-native species is native to the northwest coast of the Pacific Ocean, which has shown an expansive and colonizing period in different estuaries of the world^32–34^ since its first records in 1957 in San Francisco Bay, California^35^ and in 1979 in southeastern Australia, Newcastle^36^. Its rapid, continuous and expansive growth mode shows the colonizing success that this new exotic species is having in establishing itself in the Guadalquivir estuary, now one of the dominant species in the estuary. From the third year onwards, this introduction explains that the greatest interannual variability in the community structure has occurred between the first two years and the rest of the study period.

### Drivers of the inter-and intra-annual variations of decapod crustacean assemblage

In estuarine ecosystems of temperate regions, where temperature and salinity usually follow a similar seasonal pattern, both parameters are considered to be responsible for the temporal variations observed in their decapod crustacean communities, making it difficult to discriminate the role played by each of these variables in the seasonal changes of estuarine communities. However, in Mediterranean estuaries, such as the Guadalquivir, during dry years salinity and temperature do not evolve in parallel, which allows us to partially discriminate the effect of one or the other variable.

When comparing the changes in the community in rainy winters, when salinity decreases, with dry winters, when salinity remains practically unchanged, it is observed that in both cases the total abundance of decapod crustaceans decreases, as also occurs with other components of the estuarine fauna^1^, being, therefore, temperature, and not salinity, the one that best explains these changes in density. In other words, the seasonal variation of temperature in estuarine waters seems to be the main determinant of temporal variations in the composition and structure of estuarine communities, since it influences the activity of organisms and their growth and reproductive rates^37^.

On the other hand, the importance of salinity in the temporal changes observed in estuarine faunal composition is not the same in dry and rainy years. In hydrologically dry years, although the thermal regime of the estuarine water is not altered, the permanence of these species in the estuary, especially of *C. crangon*, is prolonged in time and, consequently, the temporal variations of the community are less than in rainy years. In other words, although temperature drives the temporal cyclical behaviour of the community, salinity acts as a complementary factor, especially in the case of marine species, affecting their permanence in the estuary^13,38^, which in the area studied particularly marks the differences in community structure. Thus, when in winter occurred high freshwater discharges, the number of species and their density decreases notably, with a greater separation between the winter samples and those of the rest of the year (Fig. 4: years 1 and 4). On the other hand, even if the freshwater discharges are not very high, when they occur in the warm period, the disturbance of the community can be greater, as occurred in the second year of the study (June 1998–May 1999). That year occurred the Aznalcóllar disaster, when extraordinary freshwater releases were made during the summer in order to dilute the effect of the polluting spill^39^. This caused a significant decrease in the density of *C. crangon*, a later entry of *P. kerathurus* and *P. serratus*, and a substantial increase of *P. longirostris* in the lower estuary, resulting in a temporarily "chaotic" cycle (Fig. 4). In contrast, in drier and therefore more saline years there is an increase in mean annual specific richness (1999, 2005 and 2006), mainly due to the influx of accidental marine species. Therefore, salinity seems to be responsible for certain interannual changes in community composition and structure.

## Material and methods

### Study area

**Figure 5:**
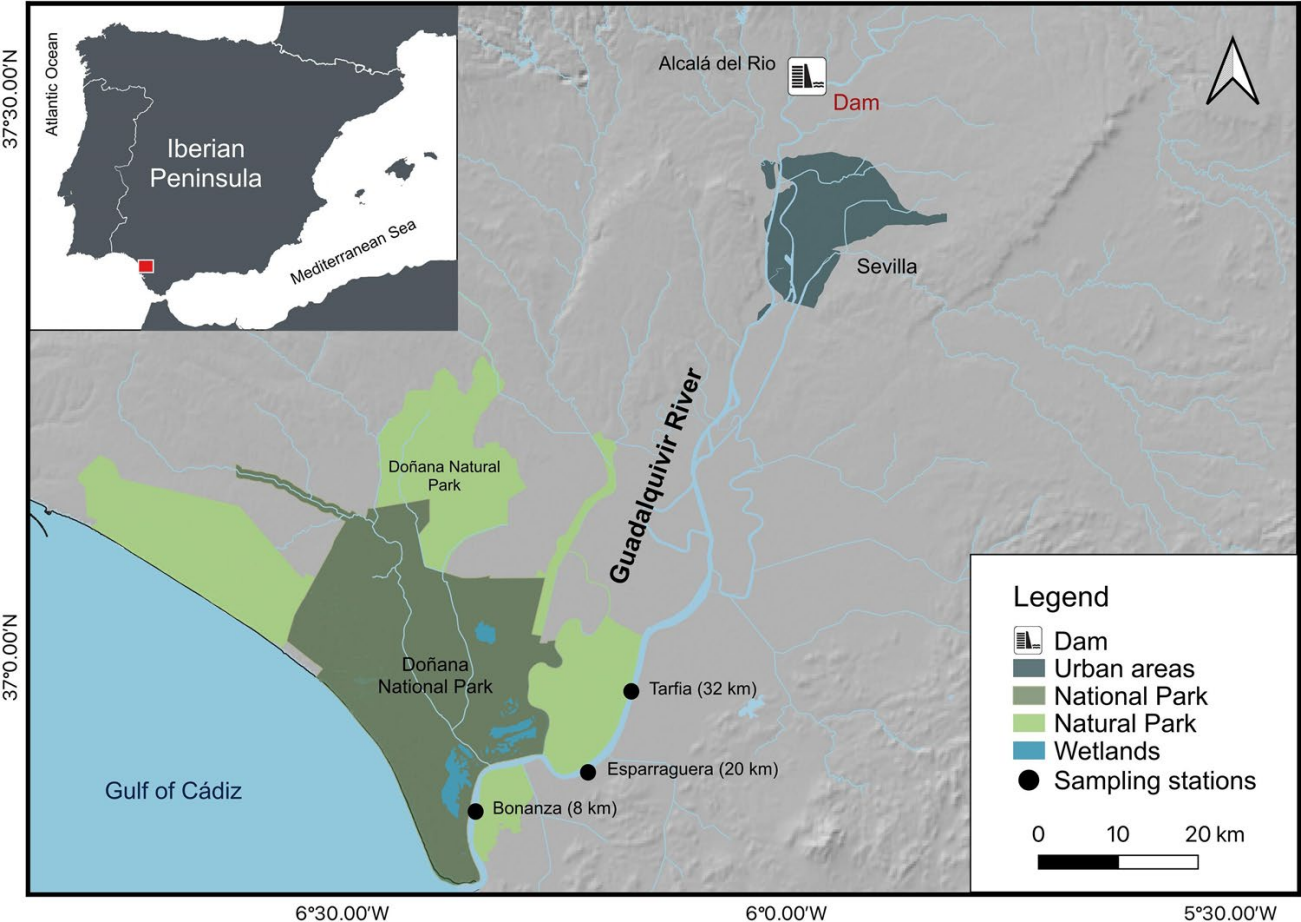
Map of the Guadalquivir estuary (Southwest Spain), indicating the locations of three sampling sites during various years from June 1997 to December 2006 (the maps were generated using QGIS 3.34 (http://qgis.osgeo.org), and WGS 84 as Coordinate Reference System).

The Guadalquivir River Basin is located within the Mediterranean-climate region (southwest Spain). However, the river empties into the Atlantic Ocean and tidal influence extends up to the Alcalá del Río dam (at 110 km from the river mouth). The freshwater inflow to the estuary is totally regulated by the dam and the estuary is a well-mixed and tidally dominated system^40^, with a longitudinal salinity gradient that shows both long-term (seasonal and inter-year) and short-term (tidal and dam management-related) displacement along the river course^1^. The area situated seaward from the isohaline 5 acts as nursery grounds for marine species^18^. On average, the isohaline 5 boundary (between the oligohaline and mesohaline zones) is situated 25 km and 35 km upstream from the river mouth at low tide and high tide, respectively; while the isohaline 18 boundary (between the mesohaline and polyhaline zones) is situated 5 km and 15 km upstream at low tide and high tide^18^. Three sampling sites were selected within this area: Tarfía (mean salinity ± standard deviation: 5.6 ± 3.6), La Esparraguera (11.1 ± 6.6) and Bonanza (21.2 ± 9.6) situated at 32, 20 and 8 km from the river’s mouth, respectively (Fig. 5).

### Data sets

Biological data analysed in this study correspond to samples collected monthly from June 1997 to December 2006 at each new moon, from a boat anchored where the water column depth was approximately 3 m at low tide. Four passive hauls were carried out at each sampling station using three 10 m long nets (net opening: 2.5 m wide and 3 m long; mesh size: 1 mm) working in parallel during the first 2 h of the diurnal and nocturnal flood and ebb tides. The total catch was emptied into a calibrated container and its volume was estimated. A subsample (thirteen litres) of the collected material, or the total volume when the catch was smaller, was randomly sampled using a calibrated beaker. In the laboratory, nektonic and hyperbenthic (fish, decapod and mysid crustaceans) organisms were sorted into species and counted. The exotic invasive species *P. macrodactylus* was identified following the illustrated key by González-Ortegón and Cuesta^41^.

Water temperature (°C), salinity and turbidity (NTU) were measured in the field at the start of each haul. The current speed during sampling was estimated with a digital flowmeter (HYDRO-BIOS®, 438 110) placed near the nets. Freshwater discharges from the Alcalá del Río dam were obtained from the Regional River Authority (“Confederación Hidrográfica del Guadalquivir”) database (http://www.juntadeandalucia.es/agenciadelagua/saih/DatosHistoricos.aspx). Rainfall in the estuarine area was obtained from the meteorological station of “El Palacio” located at Doñana National Park (http://www-rbd.ebd.csic.es/Seguimiento/mediofisico.htm) and rainfall at basin level was obtained by averaging rainfall at ten locations upstream the dam (for more details see study^42^).

### Data analysis

Since the present study focused on the identification of temporal variability changes in the nektonic community, the spatial variability of all recorded variables was removed by averaging discrete data into monthly means.

As a measure of the riverine inflow, freshwater volumes discharged from the dam to the estuary during the week (V9) or month (V28) before each sampling were estimated by adding daily freshwater flow (m^3^ s^−1^) values recorded during each of those periods; local rainfall during the week (R9) or month (R28) before each sampling date, were similarly estimated.

Previous studies found that in the Guadalquivir estuary, mysids may be present in up to 84% of the decapod crustacean guts^26^. Thus, their estuarine abundance is used in this study as a rough approximation of prey availability for the nekton.

Decapod crustaceans and mysids densities are given as the number of individuals per volume of filtered water. Constancy is the relative consistency of occurrence of a species throughout a community (number of samples where the species is found, divided by the number of samples × 100). Species were ranked according to their constancy (values ranging from 1.0 = a species that was present in all samples to 0.0 = a species that was not present) from the highest to the lowest. Decapod crustacean diversity was described for each sampling occasion on the basis of species richness (D), using Margalef’s formula^43^, and of Shannon–Wiener (Hʹ) and evenness (Jʹ) indices, following Pielou^44^. Decapod crustaceans community was also analysed according to the abundance and number of species of three ecological guilds (adapted from Elliot^45^): estuarine species (ES), species that are capable of completing their entire life cycle within the estuary; marine migrants (MM), species that spawn at sea and enter estuaries in large numbers as juveniles; and straggler species (SS), species that spawn at sea or at freshwater and enter estuaries in low numbers.

Temporal changes in the decapod crustacean community structure were also analysed following a multivariate approach. According to Clarke and Warwick^46^, rare species (present in no more than 3% of the collected samples and in less than 18% of sampling campaigns; see Table 1) were removed prior to multivariate analyses. Non-metric multidimensional scaling (nMDS) ordination of samples was carried out using the Bray–Curtis similarity measure calculated on the fourth-root transformed abundances. As a clear annual cyclicity was found, short-term effects of the freshwater input were also assessed on additional nMDS ordinations and similarity analysis, carried out after grouping samples from the 10-year survey by periods of two subsequent months. This temporal grouping allows analysing the effects of freshwater inputs on the nekton avoiding the influence of the seasonal trend (mentioned above) but with a number of samples suitable to perform statistical testing.

Cross-correlation analysis was used to ascertain individual correlation between environmental variables and the univariate measures of the decapod crustacean community structure.

The role of the environmental variables on the temporal multivariate changes observed in the nektonic community was statistically assessed by using the BEST analysis: individual correlations between each available environmental variable and the nMDS ordination of samples was estimated with the BIOENV algorithm; the best combination of variables in explaining the observed community changes with a stepwise search of variables was obtained with the BVSTEP algorithm. Significance of the correlation coefficients obtained was ascertained by random permutation tests.

BEST, nMDS, RELATE, ANOSIM analyses and permutation tests were carried out using the PRIMER v6 software package^47^.

## Data Availability

The datasets generated during and/or analysed during the current study are available from the corresponding author on reasonable request.
